# THE EFFECT OF DEPRESSION AND SUBJECTIVE HEALTH CONDITION ON INTRA-INDIVIDUAL COGNITIVE VARIABILITY

**DOI:** 10.1093/geroni/igac059.2948

**Published:** 2022-12-20

**Authors:** Shayne Lin, Rebecca Allen

**Affiliations:** University of Alabama, Tuscaloosa, Alabama, United States; University of Alabama, Tuscaloosa, Alabama, United States

## Abstract

Intra-individual cognitive variability (IICV) is defined as variability in performance across cognitive domains during neuropsychological assessment. High cognitive variability (i.e., IICV) is associated with Alzheimer’s disease diagnosis and mortality among older adults (Anderson et al., 2016; Watermeyer et al., 2021; Vaughan et al., 2013). To date, no research has explored, to the author’s knowledge, the effect of depression and physical health on IICV among older adults. The current investigation used the nationally represented Health and Retirement Study (HRS) data set and explored the effect of depression, measured by Center for Epidemiological Studies Depression (CESD), and subjective health, rated on a 5-point Likert scale, on IICV. Data from 39,800 participants and 11 time points were analyzed. IICV was calculated by the individual t score standard deviation divided by the individual mean t score. Dependent variables were centered before being entered into a multilevel model that took time of data collection into consideration. With multilevel modelling, after controlling for demographics (i.e., gender, race, ethnicity, and age), both depression, b = 0.003, SE = 0.0001, p < .001, and subjective health condition, b = 0.006, SE = 0.0002, p < .001, were associated with IICV. This study has implications about the use of IICV in neuropsychological assessment with older adults. In addition to one’s cognitive functioning, both depression and physical health have direct effect on IICV, which may be more than merely a cognitive marker for Alzheimer’s disease as researchers have previously found.

